# Pretreatment Neutrophil-to-Lymphocyte Ratio Can Predict the Prognosis in Bladder Cancer Patients Who Receive Gemcitabine and Nedaplatin Therapy

**DOI:** 10.1155/2016/9846823

**Published:** 2016-09-08

**Authors:** Shinji Ohtake, Takashi Kawahara, Ryo Kasahara, Hiroki Ito, Kimito Osaka, Yusuke Hattori, Jun-ichi Teranishi, Kazuhide Makiyama, Nobuhiko Mizuno, Susumu Umemoto, Yasuhide Miyoshi, Noboru Nakaigawa, Hiroshi Miyamoto, Masahiro Yao, Hiroji Uemura

**Affiliations:** ^1^Department of Urology, Yokohama City University Graduate School of Medicine, Yokohama, Japan; ^2^Departments of Urology and Renal Transplantation, Yokohama City University Medical Center, Yokohama, Japan; ^3^Department of Urology, Yokohama Sakae Kyosai Hospital, Yokohama, Japan; ^4^Department of Urology, Hiratsuka Kyosai Hospital, Hiratsuka, Japan; ^5^Departments of Pathology and Urology, Johns Hopkins University School of Medicine, Baltimore, MD, USA

## Abstract

*Introduction and Objectives*. Neutrophil-to-lymphocyte ratio (NLR) has been suggested to be a simple marker of the systemic inflammatory response in critical care patients. We previously assessed the utility of NLR as a biomarker to predict tumor recurrence and cancer death in bladder cancer patients who underwent radical cystectomy. In this study, we evaluated the prognostic impact of NLR in bladder cancer patients who received gemcitabine and nedaplatin (GN) chemotherapy.* Methods*. A total of 23 patients who received GN chemotherapy for advanced bladder cancer were enrolled in this study. The cut-off point of NLR according to the sensitivity and specificity levels was derived from the area under receiver operator characteristics (AUROC) curve plotted for disease progression or overall mortality.* Results*. The NLR cut-off point was determined as 4.14 for both tumor progression and overall mortality. Median progression-free survival (PFS)/overall survival (OS) in the higher NLR group (NLR ≥ 4.14) and lower NLR group (NLR < 4.14) were 194/468 days versus 73/237 days, respectively. Kaplan-Meier analysis showed that higher NLR significantly correlated with poorer PFS (*p* = 0.011) and OS (*p* = 0.045).* Conclusions*. NLR may serve as a new biomarker to predict responses to GN-based chemotherapy in advanced bladder cancer patients and/or their prognosis.

## 1. Introduction

Cisplatin alone, gemcitabine and cisplatin (GC), and methotrexate, vinblastine, doxorubicin, and cisplatin (M-VAC) have evolved as the standard first-line systemic therapy for recurrent or metastatic urothelial carcinoma (UC). However, its serious dose-limiting adverse effects include considerable renal toxicity, marked emesis, and neurotoxicity. Its nephrotoxic properties particularly make it unsuitable for patients with renal dysfunction. Indeed, UC is usually seen in the elderly, and due to age-associated impairment in the renal function and performance status, approximately 30–50% of patients are ineligible for cisplatin-based chemotherapy [1]. Instead, nedaplatin, a second-generation platinum complex with lower renal and gastrointestinal toxicities than cisplatin, can be used in patients with marginal renal function [2].

Neutrophil-to-lymphocyte ratio (NLR) has been suggested as a simple marker of the systemic inflammatory response in critical care patients [3]. NLR can be easily calculated from routine complete blood counts in the peripheral blood [4, 5]. It has also been reported to be an independent prognosticator for some solid malignancies including bladder cancer [4–13].

We previously assessed the utility of NLR as a biomarker to predict tumor recurrence and cancer death in bladder cancer patients who underwent radical cystectomy [14]. In the current study, we investigated whether NLR could predict the prognosis of bladder cancer patients who received gemcitabine and nedaplatin (GN) chemotherapy.

## 2. Materials and Methods

### 2.1. Patients

A total of 23 patients (17 men and 6 women) with measurable lesions were treated with GN chemotherapy for their advanced bladder UC at our institutions from 2005 to 2014. Of these patients, 4 underwent radical cystectomy prior to GN therapy. The mean age was 63.0 years (range 46–74), the mean creatinine clearance was 80.5 mL/min (range 43–157.1), and the mean follow-up period was 11.5 months (range 2.3–29.8). Written informed consent was obtained from all patients and the institutional review board approved this study.

### 2.2. Drug Administration and Evaluation of Responses

Patients received gemcitabine 1,000 mg/m^2^ on days 1 and 8 plus nedaplatin 80 or 100 mg/m^2^ on day 1. Dose modification was allowed depending on the patient's condition, renal function, or bone marrow suppression. Twelve patients received at least 3 cycles of GN chemotherapy, whereas the remaining 10 received 1 or 2 cycles. Tumor response was assessed according to the Response Evaluation Criteria in Solid Tumor (RECIST). Toxicity was evaluated according to the Common Terminology Criteria for Adverse Events (CTCAE) ver. 3.0.

### 2.3. Clinical and Laboratory Assessments

Complete blood cell counts (CBCs) were performed, and NLR was calculated using the neutrophil and lymphocyte counts obtained on the same day or a few days before the initial chemotherapy. We determined the cut-off point of the NLR based on the sensitivity and specificity levels derived from the area under receiver operator characteristics (AUROC) curve plotted using disease progression or overall mortality.

### 2.4. Statistical Analysis

The patient characteristics and pretreatment factors were analyzed using the Mann-Whitney *U* test and chi-square test, respectively. The Kaplan-Meier curve was used to estimate the progression-free survival (PFS) and overall survival (OS). The survival duration was defined as the time between the date of installation of GN chemotherapy and the time of tumor progression or death. The log-rank test was performed for comparison of two groups. All statistical analyses were performed using the GraphPad Prism software program (GraphPad Software, La Jolla, CA, USA). *p* < 0.05 was considered to be statistically significant.

## 3. Results

### 3.1. Patients

Of 23 patients, complete response (CR) and partial response (PR) were obtained in 2 (8.7%) and 3 (13.0%) patients, respectively. The median PFS and OS were 147 days and 396 days, respectively. Grade 3 or 4 anemia, thrombocytopenia, and neutropenia were observed in 10 (43.5%), 10 (82.6%), and 21 (91.3%) patients, respectively. None of these patients died of adverse effects of GN therapy.

### 3.2. The NLR Cut-Off Value

**Figure 1:**
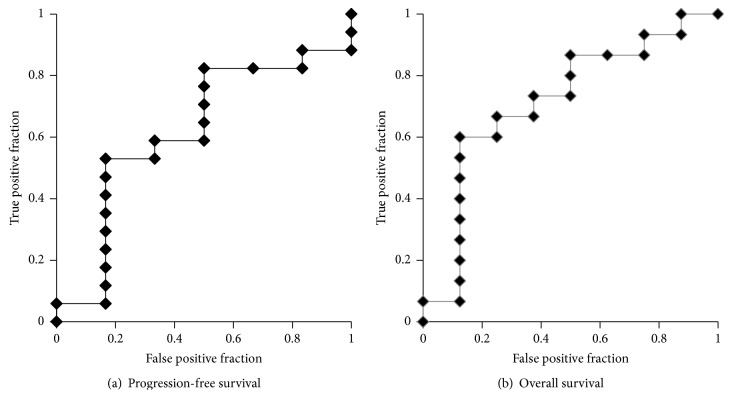
The AUROC for NLR: (a) PFS and (b) OS.

Based on the AUROC curve, the NLR cut-off point was determined to be 4.14 for both PFS (AUROC: 0.618) and OS (AUROC: 0.717) [Figure 1]. Clinicopathological characteristics of the 23 patients are summarized in Table 1. There were no statistically significant differences in the baseline characteristics between high (≥4.14) and low (<4.14) NLRs.

### 3.3. NLR and Patient Outcomes

**Figure 2:**
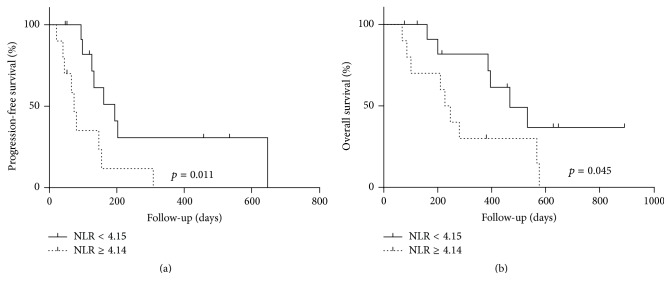
The association between NLR and patient outcomes: (a) PFS and (b) OS.

We compared PFS and OS in patients with high versus low NLRs. Kaplan-Meier analysis showed that higher NLR strongly correlated with the risks of disease progression (*p* = 0.006; Figure 2(a)) and mortality (*p* = 0.045; Figure 2(b)).

## 4. Discussion

Although advances in chemotherapy have improved the survival of patients with recurrent or metastatic UC, a portion of patients still die within a few months of disease progression. Therefore, more useful and reliable biomarkers that provide additional prognostic information are needed. CBCs are typically examined during the clinical check-up, and the NLR can be applied to all patients virtually either before or after surgery/medical treatment. We previously reported NLR as an independent prognosticator in men presenting with metastatic prostate cancer as well as in bladder cancer patients who received radical cystectomy [14]. Indeed, NLR has been shown to be a prognostic factor in patients with bladder cancer [12, 15–19]. On the other hand, the association between NLR and tumor progression remains controversial [12, 15–19]. Several studies have shown a higher NLR to predict a worse prognosis in bladder cancer patients [16, 18–20], whereas others have concluded that NLR is not strongly correlated with OS [12, 15–18]. In the current study, higher NLR significantly correlated with a poorer prognosis in patients who received GN chemotherapy for their advanced bladder cancer.

In addition to cisplatin, various anticancer platinum complexes have been developed. Carboplatin, a cisplatin analogue, has been shown to exhibit improved toxicity and favorable antitumor effects, resulting in response rates of 18.4% for upper urinary tract UC [20]. Additionally, nedaplatin was developed as a second-generation platinum complex with lower renal and gastrointestinal toxicities compared with cisplatin [21]. Sasaki et al. demonstrated that the pharmacokinetic behavior of nedaplatin was similar to that of carboplatin but is strikingly different from that of cisplatin. Cisplatin easily binds to serum proteins, resulting in a smaller percentage of platinum excreted into the urine after infusion compared with nedaplatin or carboplatin [22]. Matsumoto et al. showed greater activity of GN therapy against lung cancer models than the activity of a combination of gemcitabine with cisplatin or carboplatin [23]. In our institution, we have used nedaplatin-based chemotherapy for high-grade UC and have demonstrated good responses, with the median PFS and OS times of 147 and 396 days, respectively [2, 24].

There are several limitations associated with this study, including selection bias and missing data for some of the variables due to its retrospective nature. However, this study may provide supportive data for other studies as well as future prospective studies. Another potential limitation is that we did not determine the mechanism of NLR for bladder cancer progression. Previous studies showed a correlation between NLR as a marker of systemic inflammation in cancer patients and patient outcomes.

In conclusion, we demonstrated that NLR might be a new biomarker to predict the prognosis of advanced bladder cancer in patients undergoing GN chemotherapy.

## Figures and Tables

**Table 1:** Clinicopathological characteristics of the patients.

	Total	NLR < 4.14	NLR ≧ 4.14	*p* value
	(*n* = 23)	(*n* = 9)	(*n* = 14)	
Age (years)				
<65	11 (47.6%)	4 (44.4%)	7 (50.0%)	0.566
≥65	12 (52.4%)	5 (55.6%)	7 (50.0%)	
Gender				
Female	6 (26.1%)	4 (44.4%)	2 (14.3%)	0.131
Male	17 (73.9%)	5 (55.6%)	12 (85.7%)	
Creatinine clearance (mL/min)				
<60	3 (13.0%)	2 (22.2%)	1 (7.1%)	0.332
≥60	20 (87.0%)	7 (77.8%)	13 (92.9%)	
Clinical lymph node metastasis				
Yes	19 (82.6%)	8 (88.9%)	11 (78.6%)	0.483
No	4 (17.4%)	1 (11.1%)	3 (21.4%)	
Neoadjuvant chemotherapy				
Yes	4 (17.4%)	3 (33.3%)	1 (7.1%)	0.147
No	19 (82.6%)	6 (66.7%)	13 (92.9%)	
Clinical T stage				
≤2	6 (26.1%)	2 (22.2%)	4 (28.6%)	0.565
≥3	17 (73.9%)	7 (77.8%)	10 (71.4%)	
